# Supplementary material for: End Sequence Analysis Toolkit (ESAT) expands the extractable information from single-cell RNA-seq data
Source: Genome Res. 2016 Oct;26(10):1397–410. doi: 10.1101/gr.207902.116 (PMC5052061; doi:10.1101/gr.207902.116)
Supplement: Supplemental Material [file supp_gr.207902.116_Supp_Materials.tar.gz › ESAT-esat_v0.1/doc/html/classumms_1_1core_1_1annotation_1_1_abstract_annotation.html]

ESAT: umms.core.annotation.AbstractAnnotation Class Reference


ESAT


- Main Page
- Related Pages
- Packages
- Classes
- Files

- Class List
- Class Index
- Class Hierarchy
- Class Members

All Classes Namespaces Files Functions Variables Enumerator Pages

- umms
- core
- annotation
- AbstractAnnotation

Public Member Functions |
Static Public Member Functions |
Static Public Attributes |
List of all members

umms.core.annotation.AbstractAnnotation Class Referenceabstract

Inheritance diagram for umms.core.annotation.AbstractAnnotation:

[legend]

Collaboration diagram for umms.core.annotation.AbstractAnnotation:

[legend]

|  |  |
| --- | --- |
| Public Member Functions | |
| int | size () |
|  | |
| int | getSize () |
|  | |
| int | getMidpoint () |
|  | |
| Strand | getStrand () |
|  | |
| boolean | hasOrientation () |
|  | |
| boolean | isNegativeStrand () |
|  | |
| String | getChr () |
|  | |
| int | getOrientedStart () |
|  | |
| int | getOrientedEnd () |
|  | |
| int | getLengthOnReference () |
|  | |
| int | getReferenceCoordinateAtPosition (int positionInAnnotation) |
|  | |
| int | getPositionAtReferenceCoordinate (int referenceCoordinate) |
|  | |
| void | setOrientation (char orientation) |
|  | |
| void | expand (int deltaStart, int deltaEnd) |
|  | |
| Annotation | trim (int deltaStart, int deltaEnd) |
|  | |
| boolean | fullyContains (Annotation other) |
|  | |
| boolean | overlaps (Annotation other) |
|  | |
| boolean | overlaps (Annotation other, int buffer) |
|  | |
| boolean | overlapsStranded (Annotation other) |
|  | |
| boolean | overlaps (Annotation other, boolean considerOrientation) |
|  | |
| boolean | overlaps (Collection<?extends Annotation > others) |
|  | |
| boolean | overlaps (Collection<?extends Annotation > others, int buffer) |
|  | |
| String | toUCSC () |
|  | |
| String | toBED () |
|  | |
| final String | getFullInfoString () |
|  | |
| String | toBED (int r, int g, int b) |
|  | |
| String | toShortBED () |
|  | |
| String | toBEDGraph () |
|  | |
| int | getDistanceTo (Annotation other) |
|  | |
| String | toCigar () |
|  | |
| List< Annotation > | intersect (List<?extends Annotation > others) |
|  | |
| int | compareTo (Annotation other) |
|  | |
| int | compareToAnnotation (Annotation b) |
|  | |
| int | compareToAnnotation (Annotation b, boolean useOrientation) |
|  | |
| boolean | equals (Annotation a) |
|  | |
| boolean | equals (Annotation a, boolean useOrientation) |
|  | |
| int | getSAMStart () |
|  | |
| int | getSAMEnd () |
|  | |
| Public Member Functions inherited from umms.core.annotation.Annotation | |
| int | getStart () |
|  | |
| int | getEnd () |
|  | |
| String | getReferenceName () |
|  | |
| String | getName () |
|  | |
| Strand | getOrientation () |
|  | |
| int | numBlocks () |
|  | |
| List<?extends Annotation > | getBlocks () |
|  | |
| List<?extends Annotation > | getBlocks (boolean oriented) |
|  | |
| int | length () |
|  | |
| double | getScore () |
|  | |
| boolean | isUnoriented () |
|  | |
| int | getReferenceCoordinateAtPosition (int positionInAnnotation, boolean ignoreOrientation) |
|  | |
| int | getPositionAtReferenceCoordinate (int referenceCoordinate, boolean ignoreOrientation) |
|  | |
| void | setStart (int start) |
|  | |
| void | setEnd (int end) |
|  | |
| void | setOrientation (Strand orientation) |
|  | |
| void | setOrientedStart (int orientedStart) |
|  | |
| void | setOrientedEnd (int orientedEnd) |
|  | |
| void | setReferenceName (String refName) |
|  | |
| void | setName (String name) |
|  | |
| void | setScore (double score) |
|  | |
| void | shift (int delta) |
|  | |
| void | moveToCoordinate (int coordinateInReference) |
|  | |
| Annotation | copy () |
|  | |
| List< Annotation > | disect (Annotation a) |
|  | |
| List< Annotation > | disect (List<?extends Annotation > disectors) |
|  | |
| Annotation | minus (Annotation other) |
|  | |
| Annotation | minus (Collection<?extends Annotation > others) |
|  | |
| boolean | overlaps (Annotation other, int buffer, boolean considerOrientation) |
|  | |
| int | getOverlap (Annotation other) |
|  | |
| boolean | contains (Annotation other) |
|  | |
| Annotation | union (Annotation other) |
|  | |
| Annotation | intersect (Annotation other) |
|  | |
| void | stitchTo (Annotation next) |
|  | |
| Annotation | complement () |
|  | |
| Collection<?extends Annotation > | getSpliceConnections () |
|  | |

|  |  |
| --- | --- |
| Static Public Member Functions | |
| static Strand | getStrand (String orientation) |
|  | |
| static Strand | getStrand (char orientation) |
|  | |

|  |  |
| --- | --- |
| Static Public Attributes | |
| static final int | MAX\_DISTANCE = 1000000000 |
|  | |

## Detailed Description

Author
:   engreitz This abstract class contains generic implementations of Annotation functions that rely only on other Annotation functions.

## Member Function Documentation

|  |  |  |  |  |  |
| --- | --- | --- | --- | --- | --- |
| int umms.core.annotation.AbstractAnnotation.compareTo | ( | Annotation | *other* | ) |  |

Here is the call graph for this function:

|  |  |  |  |  |  |
| --- | --- | --- | --- | --- | --- |
| int umms.core.annotation.AbstractAnnotation.compareToAnnotation | ( | Annotation | *b* | ) |  |

Should behave like compareTo for a basic annotation

Parameters
:   |  |  |
    | --- | --- |
    | b |  |

Returns

Implements umms.core.annotation.Annotation.

Here is the caller graph for this function:

|  |  |  |  |
| --- | --- | --- | --- |
| int umms.core.annotation.AbstractAnnotation.compareToAnnotation | ( | Annotation | *b*, |
|  |  | boolean | *useOrientation* |
|  | ) |  |  |

Here is the call graph for this function:

|  |  |  |  |  |  |
| --- | --- | --- | --- | --- | --- |
| boolean umms.core.annotation.AbstractAnnotation.equals | ( | Annotation | *a* | ) |  |

Implements umms.core.annotation.Annotation.

Here is the caller graph for this function:

|  |  |  |  |
| --- | --- | --- | --- |
| boolean umms.core.annotation.AbstractAnnotation.equals | ( | Annotation | *other*, |
|  |  | boolean | *useOrientation* |
|  | ) |  |  |

Test whether two annotations are equal regardless of whether the orientation are the same

Parameters
:   |  |  |
    | --- | --- |
    | other | The annotation to test equality to |
    | useOrientation | true=ensure strands match false=OK if strands dont match |

Returns

Implements umms.core.annotation.Annotation.

Here is the call graph for this function:

|  |  |  |  |
| --- | --- | --- | --- |
| void umms.core.annotation.AbstractAnnotation.expand | ( | int | *deltaStart*, |
|  |  | int | *deltaEnd* |
|  | ) |  |  |

Extends the annotation by delta on the unOriented Start and end.

Implements umms.core.annotation.Annotation.

Here is the call graph for this function:

|  |  |  |  |  |  |
| --- | --- | --- | --- | --- | --- |
| boolean umms.core.annotation.AbstractAnnotation.fullyContains | ( | Annotation | *annotation* | ) |  |

Test whether the annotation is fully contained in this

Parameters
:   |  |  |
    | --- | --- |
    | annotation |  |

Returns

Implements umms.core.annotation.Annotation.

Here is the call graph for this function:

|  |  |  |  |  |
| --- | --- | --- | --- | --- |
| String umms.core.annotation.AbstractAnnotation.getChr | ( |  | ) |  |

Returns
:   name of the reference sequence

Implements umms.core.annotation.Annotation.

Here is the call graph for this function:

Here is the caller graph for this function:

|  |  |  |  |  |  |
| --- | --- | --- | --- | --- | --- |
| int umms.core.annotation.AbstractAnnotation.getDistanceTo | ( | Annotation | *other* | ) |  |

Calculates the distance to the another genomic annotation.

Returns
:   0 if the annotations overlap or the minimum between the edges otherwise.

Implements umms.core.annotation.Annotation.

Here is the call graph for this function:

|  |  |  |  |  |
| --- | --- | --- | --- | --- |
| final String umms.core.annotation.AbstractAnnotation.getFullInfoString | ( |  | ) |  |

Returns
:   A string with no whitespace that includes all information about the annotation

Implements umms.core.annotation.Annotation.

Here is the call graph for this function:

|  |  |  |  |  |
| --- | --- | --- | --- | --- |
| int umms.core.annotation.AbstractAnnotation.getLengthOnReference | ( |  | ) |  |

Returns
:   The distance between the start of the first block and end of the last on the reference sequence (including spaces between blocks)

Implements umms.core.annotation.Annotation.

Here is the call graph for this function:

Here is the caller graph for this function:

|  |  |  |  |  |
| --- | --- | --- | --- | --- |
| int umms.core.annotation.AbstractAnnotation.getMidpoint | ( |  | ) |  |

This will return the position in the center of this annotation taking into account the blocked structure

Returns

Implements umms.core.annotation.Annotation.

Here is the call graph for this function:

|  |  |  |  |  |
| --- | --- | --- | --- | --- |
| int umms.core.annotation.AbstractAnnotation.getOrientedEnd | ( |  | ) |  |

Returns
:   the 3-prime end of the annotation, considering its orientation or strand

Implements umms.core.annotation.Annotation.

Here is the call graph for this function:

|  |  |  |  |  |
| --- | --- | --- | --- | --- |
| int umms.core.annotation.AbstractAnnotation.getOrientedStart | ( |  | ) |  |

Returns
:   the 5-prime end of the annotation, considering its orientation or strand

Implements umms.core.annotation.Annotation.

Here is the call graph for this function:

|  |  |  |  |  |  |
| --- | --- | --- | --- | --- | --- |
| int umms.core.annotation.AbstractAnnotation.getPositionAtReferenceCoordinate | ( | int | *referenceCoordinate* | ) |  |

Parameters
:   |  |  |
    | --- | --- |
    | referenceCoordinate | 0-based reference coordinate |

Returns
:   0-based position in the Annotation, considering orientation

Implements umms.core.annotation.Annotation.

|  |  |  |  |  |  |
| --- | --- | --- | --- | --- | --- |
| int umms.core.annotation.AbstractAnnotation.getReferenceCoordinateAtPosition | ( | int | *positionInAnnotation* | ) |  |

Parameters
:   |  |  |
    | --- | --- |
    | positionInAnnotation | 0-based position in the Annotation |

Returns
:   0-based reference coordinate at the specified position, considering orientation. NOTE: positionInAnnotation=0 for a negative-strand annotation will match getEnd()

Implements umms.core.annotation.Annotation.

Here is the caller graph for this function:

|  |  |  |  |  |
| --- | --- | --- | --- | --- |
| int umms.core.annotation.AbstractAnnotation.getSAMEnd | ( |  | ) |  |

Returns the start position of this window into SAM coordinate space SAM coordinates are 1-based and inclusive whereas all of our objects are 0-based exclusive

Returns

Implements umms.core.annotation.Annotation.

Here is the call graph for this function:

|  |  |  |  |  |
| --- | --- | --- | --- | --- |
| int umms.core.annotation.AbstractAnnotation.getSAMStart | ( |  | ) |  |

Returns the start position of this window into SAM coordinate space SAM coordinates are 1-based and inclusive whereas all of our objects are 0-based exclusive

Returns

Implements umms.core.annotation.Annotation.

Here is the call graph for this function:

Here is the caller graph for this function:

|  |  |  |  |  |
| --- | --- | --- | --- | --- |
| int umms.core.annotation.AbstractAnnotation.getSize | ( |  | ) |  |

Returns
:   same as size();

Implements umms.core.annotation.Annotation.

Here is the call graph for this function:

Here is the caller graph for this function:

|  |  |  |  |  |
| --- | --- | --- | --- | --- |
| Strand umms.core.annotation.AbstractAnnotation.getStrand | ( |  | ) |  |

Returns
:   orientation or strand

Implements umms.core.annotation.Annotation.

Here is the call graph for this function:

Here is the caller graph for this function:

|  |  |  |  |  |  |
| --- | --- | --- | --- | --- | --- |
| static Strand umms.core.annotation.AbstractAnnotation.getStrand | ( | String | *orientation* | ) |  |

static

|  |  |  |  |  |  |
| --- | --- | --- | --- | --- | --- |
| static Strand umms.core.annotation.AbstractAnnotation.getStrand | ( | char | *orientation* | ) |  |

static

|  |  |  |  |  |
| --- | --- | --- | --- | --- |
| boolean umms.core.annotation.AbstractAnnotation.hasOrientation | ( |  | ) |  |

Returns
:   true if strand is known

Implements umms.core.annotation.Annotation.

Here is the call graph for this function:

Here is the caller graph for this function:

|  |  |  |  |  |  |
| --- | --- | --- | --- | --- | --- |
| List<Annotation> umms.core.annotation.AbstractAnnotation.intersect | ( | List<?extends Annotation > | *annotations* | ) |  |

Returns the result of intersecting this instance with a list of annotations, with one (possibly blocked) intersection Annotation for each provided annotation

Parameters
:   |  |  |
    | --- | --- |
    | other | annotations |

Returns
:   List of intersections or empty list if none

Implements umms.core.annotation.Annotation.

|  |  |  |  |  |
| --- | --- | --- | --- | --- |
| boolean umms.core.annotation.AbstractAnnotation.isNegativeStrand | ( |  | ) |  |

Returns true if the alignment is negative stranded

Returns

Implements umms.core.annotation.Annotation.

Here is the call graph for this function:

|  |  |  |  |  |  |
| --- | --- | --- | --- | --- | --- |
| boolean umms.core.annotation.AbstractAnnotation.overlaps | ( | Annotation | *other* | ) |  |

Parameters
:   |  |  |
    | --- | --- |
    | other | GenomicAnnotation to check of overlap |

Returns
:   true if the current instance overlaps with the other one, not considering orientation

Implements umms.core.annotation.Annotation.

Here is the caller graph for this function:

|  |  |  |  |
| --- | --- | --- | --- |
| boolean umms.core.annotation.AbstractAnnotation.overlaps | ( | Annotation | *other*, |
|  |  | int | *buffer* |
|  | ) |  |  |

Parameters
:   |  |  |
    | --- | --- |
    | other | - other genomic annotation |
    | buffer | if the overlap is within buffer they will be considered overlapping even if they do not overlap within their original boundaries. |

Returns
:   true if they overlap in this extended definition, not considering orientation

Implements umms.core.annotation.Annotation.

Here is the call graph for this function:

|  |  |  |  |
| --- | --- | --- | --- |
| boolean umms.core.annotation.AbstractAnnotation.overlaps | ( | Annotation | *other*, |
|  |  | boolean | *considerOrientation* |
|  | ) |  |  |

Implements umms.core.annotation.Annotation.

Here is the call graph for this function:

|  |  |  |  |  |  |
| --- | --- | --- | --- | --- | --- |
| boolean umms.core.annotation.AbstractAnnotation.overlaps | ( | Collection<?extends Annotation > | *others* | ) |  |

Parameters
:   |  |  |
    | --- | --- |
    | other | GenomicAnnotations to check of overlap |

Returns
:   true if the current instance overlaps with the other one, not considering orientation

Implements umms.core.annotation.Annotation.

Here is the call graph for this function:

|  |  |  |  |
| --- | --- | --- | --- |
| boolean umms.core.annotation.AbstractAnnotation.overlaps | ( | Collection<?extends Annotation > | *others*, |
|  |  | int | *buffer* |
|  | ) |  |  |

Parameters
:   |  |  |
    | --- | --- |
    | other | - other genomic annotation |
    | buffer | if the overlap is within buffer they will be considered overlapping even if they do not overlap within their original boundaries. |

Returns
:   true if they overlap in this extended definition, not considering orientation

Implements umms.core.annotation.Annotation.

Here is the call graph for this function:

|  |  |  |  |  |  |
| --- | --- | --- | --- | --- | --- |
| boolean umms.core.annotation.AbstractAnnotation.overlapsStranded | ( | Annotation | *other* | ) |  |

Implements umms.core.annotation.Annotation.

Here is the call graph for this function:

|  |  |  |  |  |  |
| --- | --- | --- | --- | --- | --- |
| void umms.core.annotation.AbstractAnnotation.setOrientation | ( | char | *orientation* | ) |  |

Implements umms.core.annotation.Annotation.

|  |  |  |  |  |
| --- | --- | --- | --- | --- |
| int umms.core.annotation.AbstractAnnotation.size | ( |  | ) |  |

Returns
:   same as length()

Implements umms.core.annotation.Annotation.

Here is the call graph for this function:

Here is the caller graph for this function:

|  |  |  |  |  |
| --- | --- | --- | --- | --- |
| String umms.core.annotation.AbstractAnnotation.toBED | ( |  | ) |  |

Implements umms.core.annotation.Annotation.

Here is the caller graph for this function:

|  |  |  |  |
| --- | --- | --- | --- |
| String umms.core.annotation.AbstractAnnotation.toBED | ( | int | *r*, |
|  |  | int | *g*, |
|  |  | int | *b* |
|  | ) |  |  |

Implements umms.core.annotation.Annotation.

Here is the call graph for this function:

|  |  |  |  |  |
| --- | --- | --- | --- | --- |
| String umms.core.annotation.AbstractAnnotation.toBEDGraph | ( |  | ) |  |

Implements umms.core.annotation.Annotation.

Here is the call graph for this function:

|  |  |  |  |  |
| --- | --- | --- | --- | --- |
| String umms.core.annotation.AbstractAnnotation.toCigar | ( |  | ) |  |

Return the Annotation object cigar string

Returns

Implements umms.core.annotation.Annotation.

Here is the call graph for this function:

Here is the caller graph for this function:

|  |  |  |  |  |
| --- | --- | --- | --- | --- |
| String umms.core.annotation.AbstractAnnotation.toShortBED | ( |  | ) |  |

Implements umms.core.annotation.Annotation.

Here is the call graph for this function:

|  |  |  |  |  |
| --- | --- | --- | --- | --- |
| String umms.core.annotation.AbstractAnnotation.toUCSC | ( |  | ) |  |

Returns
:   chrX:start-end

Implements umms.core.annotation.Annotation.

Here is the call graph for this function:

Here is the caller graph for this function:

|  |  |  |  |
| --- | --- | --- | --- |
| Annotation umms.core.annotation.AbstractAnnotation.trim | ( | int | *deltaStart*, |
|  |  | int | *deltaEnd* |
|  | ) |  |  |

Trims the annotation in a strand-specific manner, that is start will end the genomic end on a negative annotation

Implements umms.core.annotation.Annotation.

Here is the call graph for this function:

Here is the caller graph for this function:

## Member Data Documentation

final int umms.core.annotation.AbstractAnnotation.MAX\_DISTANCE = 1000000000

static

---

The documentation for this class was generated from the following file:

- java/umms/core/annotation/AbstractAnnotation.java


---

Generated on Fri Aug 15 2014 15:11:07 for ESAT by  

 1.8.7
